# Nurse-Led Educational Interventions Significantly Improve Treatment Adherence and Psychosocial Outcomes in Chemotherapy Patients: A Meta-analysis

**DOI:** 10.1007/s12529-026-10442-w

**Published:** 2026-02-11

**Authors:** QiongFei Hu, JingNa Zhou, MeiDan Zhou, Lili Sun

**Affiliations:** 1 The Affiliated Yangming Hospital of Ningbo University (Yuyao People’s Hospital), Ningbo University, Ningbo, China; 2 Yuyao People’s Hospital, Ningbo University, Ningbo, China

**Keywords:** Nurse-led education, Chemotherapy adherence, Meta-analysis, Psychosocial outcomes, Oncology nursing

## Abstract

**Background:**

Patient nonadherence to chemotherapy remains a significant barrier to effective cancer treatment. Nurse-led educational interventions have been increasingly implemented to address this issue; however, the reported outcomes have been inconsistent. This meta-analysis aims to evaluate the effectiveness of nursing education in improving treatment adherence and related proxy outcomes—such as self-efficacy, symptom burden, and quality of life—among patients undergoing chemotherapy.

**Method:**

A systematic literature search was conducted across three major electronic databases—PubMed, Web of Science, and Scopus—from inception to June 2025, with additional records identified via Google Scholar. Of 194 records initially retrieved, 19 studies met the eligibility criteria and were included in the meta-analysis. Eligibility criteria encompassed studies involving adult cancer patients receiving chemotherapy, employing randomized controlled trials or quasi-experimental designs, and assessing nurse-led educational interventions on adherence outcomes. Depending on the outcome type, either standardized mean differences (SMDs) or odds ratios (ORs) were calculated using a random-effects model.

**Results:**

This meta-analysis included 1339 participants from 19 studies. For continuous outcomes, the pooled SMD was 0.89 (95% CI, 0.69–1.09), indicating a substantial positive effect of nurse-led education. For dichotomous outcomes, the pooled OR was 4.37 (95% CI, 1.32–14.49), suggesting that patients receiving educational interventions from nurses were over four times more likely to adhere to treatment. Heterogeneity was minimal (*I*^2^ = 0%), and no significant publication bias was detected. Notably, face-to-face and digital platform interventions showed significant efficacy in improving direct adherence outcomes, while all modalities positively influenced proxy outcomes such as self-efficacy and quality of life.

**Conclusion:**

Our meta-analysis suggests that nurse-led educational interventions may significantly improve treatment adherence and associated psychosocial outcomes among chemotherapy patients, although causality cannot be definitively established due to the observational nature of the included studies. These findings support the routine integration of structured nursing education into oncology care to enhance patient engagement and optimize therapeutic outcomes.

## Introduction

Adherence to chemotherapy is a key determinant of clinical outcomes [1]. However, despite advancements in oncology care, nonadherence remains widespread and is associated with increased morbidity, mortality, and healthcare expenditures. Common contributing factors include symptom burden, psychosocial distress, insufficient patient education, and inadequate communication with healthcare providers [1–3].

To address these challenges, nurse-led educational interventions have emerged as a patient-centered approach to improve adherence [4–6]. As frontline healthcare providers, nurses are uniquely positioned to deliver education that enhances treatment understanding, strengthens self-management capabilities, and encourages active patient participation [7–9]. Prior studies have emphasized the critical role of nurses in supporting adherence—particularly in oral chemotherapy—through continuous patient education, symptom monitoring, and individualized counseling [10]. Trials assessing direct adherence indicators, such as medication possession ratio, primarily involved nurse-led educational interviews, reinforcing the role of nurses in optimizing behavioral outcomes through structured education. These interventions are delivered through diverse formats, including face-to-face sessions, printed materials, and technology-assisted platforms, allowing for customization based on individual patient needs [3, 11, 12]. Multidisciplinary teams, including oncology nurses, are essential in chemotherapy to coordinate care and education, ensuring that interventions address complex patient needs and environmental factors. Empirical evidence suggests that such tailored educational approaches not only improve patient comprehension but also alleviate anxiety, enhance quality of life, and foster continuity of care [1, 13, 14]. Home chemotherapy reduces exposure to hospital-acquired infections, particularly beneficial for immunocompromised patients, and highlights the need for nurse-led education to ensure safe home-based adherence. Additionally, simplified educational strategies administered through nurse counseling have been shown to effectively address knowledge gaps and improve adherence [15].

Educational interventions should be grounded in behavioral theories such as the Theory of Planned Behavior, which links attitudes, subjective norms, and perceived behavioral control to adherence behaviors. Theoretical frameworks such as Bandura’s self-efficacy theory and the Health Belief Model provide strong conceptual support for the inclusion of these measures, as they influence health behaviors and long-term treatment engagement. The Health Belief Model provides a theoretical framework for nurse-led interventions, positing that patients’ adherence behaviors are influenced by perceived susceptibility, severity, benefits, and barriers, which can be modified through targeted education [13]. Consolidating these frameworks helps explain how education impacts adherence.

While numerous individual studies have reported positive outcomes, the overall magnitude and consistency of these effects remain uncertain due to methodological variability across studies—including differences in design, sample size, intervention modality, and outcome measurement [16–18]. Some trials have assessed direct indicators of adherence, such as medication compliance and appointment attendance [19–21]. These outcomes have been directly linked to improved clinical results, highlighting the importance of targeted educational strategies to promote adherence [22, 23]. Other studies have focused on proxy outcomes—such as self-efficacy, symptom burden, and patient satisfaction—which represent important psychosocial and behavioral determinants of adherence [24–26]. These studies consistently demonstrated that improvements in proxy outcomes, such as reduced symptom burden, correlate with enhanced adherence, supporting their use as valid indicators in interventional research.

Previous studies have reported mixed findings on nurse-led educational interventions, with variations in design and outcomes, but no meta-analysis has synthesized both direct and proxy adherence measures. Our study addresses this gap by providing a comprehensive quantitative synthesis to establish evidence-based practices. To date, no comprehensive meta-analysis has synthesized the findings from studies assessing both direct and proxy adherence outcomes. Therefore, this study aims to evaluate the overall effectiveness of nurse-led educational interventions on chemotherapy adherence and its related psychosocial indicators, with the goal of informing future development and integration of evidence-based nursing practices in oncology care.

## Methods

### Literature Search Strategy

**Fig. 1:**
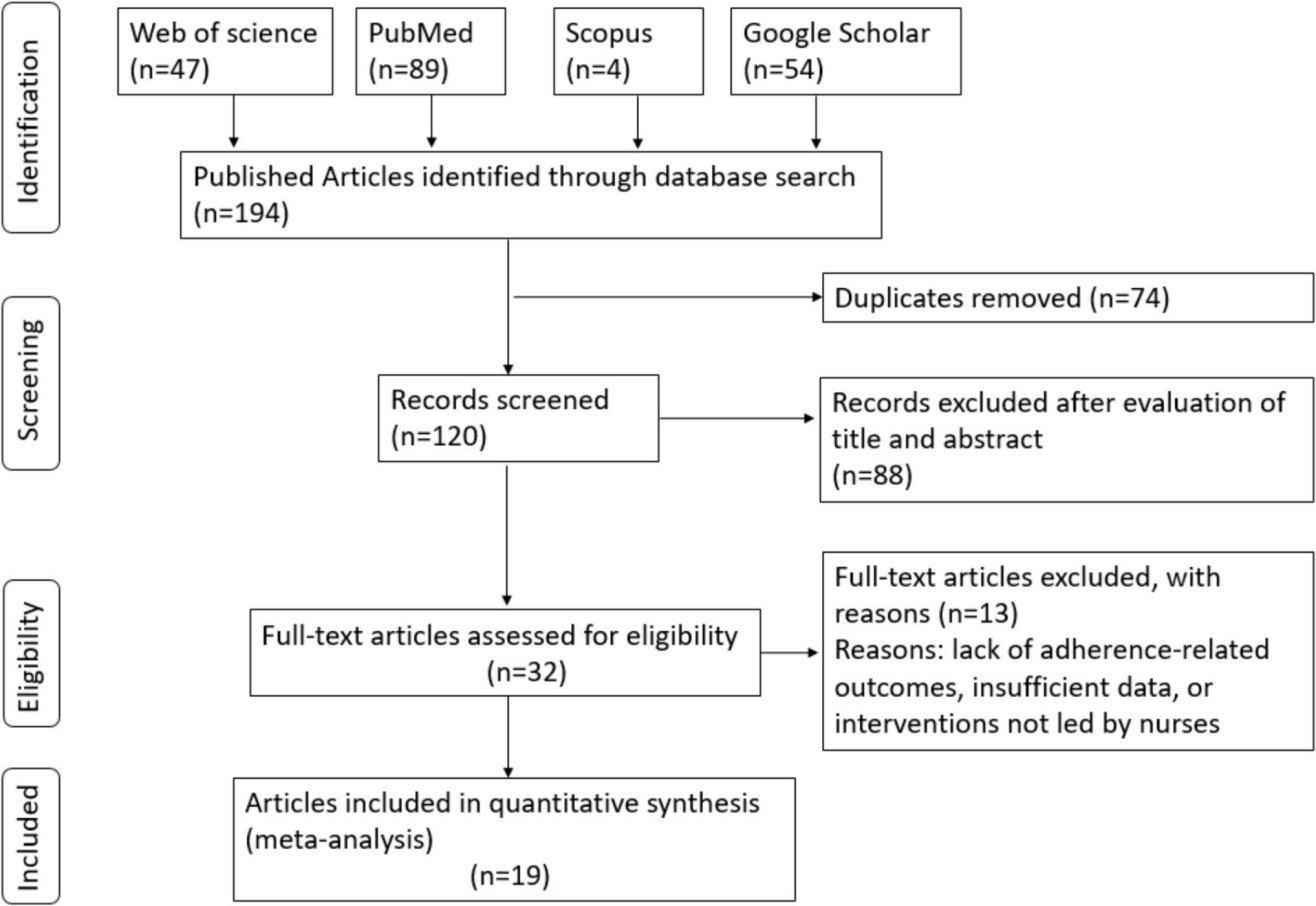
PRISMA flow diagram illustrating the study selection process. A total of 194 records were identified through systematic searches of three major electronic databases (PubMed, 89; Web of Science, 47; and Scopus, 4), supplemented by an additional 54 records from Google Scholar. After removing duplicates, 120 records remained for title and abstract screening. Of these, 88 records were excluded for not meeting inclusion criteria. Thirty-two full-text articles were assessed for eligibility, and 13 were excluded due to reasons such as lack of adherence-related outcomes, insufficient data, or interventions not led by nurses. Finally, 19 studies met all criteria and were included in both qualitative synthesis and quantitative meta-analysis

This meta-analysis was conducted in accordance with the PRISMA guidelines (Fig. 1). A comprehensive literature search was performed across three major electronic databases—PubMed, Web of Science, and Scopus—from their inception to June 2025. The search strategy incorporated both keywords and Boolean operators, using terms such as “nursing education,” “patient adherence,” “chemotherapy,” “self-efficacy,” and “quality of life.”

In PubMed, the search string was:

- nursing education AND patient adherence AND chemotherapy AND (self-efficacy OR quality of life)

Filters were applied to limit results to English-language articles and study types such as Clinical Trials, Randomized Controlled Trials, and Comparative Studies.

For Web of Science, the search used:

- (“nursing education”) AND (“patient adherence”) AND (“chemotherapy”) AND (“self-efficacy” OR “quality of life”)

Results were filtered to include only English-language articles.

In Scopus, a phrase-based search using “The Impact of Nursing Education on Treatment Adherence” was conducted.

Additionally, Google Scholar was used as a supplementary source to capture relevant studies not indexed in the primary databases. Reference lists of included studies and related reviews were manually screened to ensure completeness. Only peer-reviewed articles published in English were considered for inclusion.

Inclusion criteria:

Studies were eligible if they:

- Involved adult patients undergoing chemotherapy for cancer;
- Evaluated nursing education interventions (in-person, written, or technology-assisted formats);
- Reported treatment adherence outcomes—either direct (e.g., medication adherence, appointment attendance) or proxy (e.g., self-efficacy, symptom burden, quality of life);
- Adopted a randomized controlled trial (RCT) or quasi-experimental design;
- Provided sufficient data to calculate effect sizes.

Exclusion criteria:

- Qualitative studies, review articles, study protocols, editorials, and conference abstracts;
- Studies not reporting any adherence-related outcomes;
- Interventions not primarily delivered by nurses.

### Outcome Measures

Two categories of outcome measures were analyzed to capture both behavioral adherence and related psychosocial constructs:

- Direct adherence outcomes, including objective indicators such as medication possession ratio (MPR ≥ 90%), appointment attendance, and scores on validated adherence scales (e.g., Morisky Medication Adherence Scale);
- Proxy adherence outcomes, which reflect behavioral or emotional influences on adherence, such as self-efficacy (e.g., MASES, GSE), quality of life (e.g., EORTC QLQ-C30, FACT-B), and symptom burden (e.g., MSAS, SAS).

### Data Extraction

Two independent reviewers (L.S. and Q.H.) extracted data using a standardized data extraction form. Extracted variables included author, publication year, study design, sample size, type of nursing education intervention, adherence outcome(s), and relevant statistical data (e.g., means and standard deviations or event counts). Discrepancies were resolved through discussion and consensus.

### Statistical Analysis

All analyses were conducted using a random-effects model to account for between-study heterogeneity. For continuous outcomes, standardized mean differences (SMD) were calculated; for dichotomous outcomes, odds ratios (OR) were used. Both were reported with corresponding 95% confidence intervals (CI). Statistical heterogeneity was assessed using the *I*^2^ statistic, with values above 50% indicating moderate to high heterogeneity.

Forest plots were used to visually display individual and pooled effect sizes. Funnel plots were generated to assess potential publication bias. All statistical computations were performed using Python (for meta-analytical calculations), and OriginPro 2019b was used for figure generation and visualization.

## Results

### Overall Study Characteristics

The meta-analysis included diverse patient populations, primarily adults with various cancer types undergoing chemotherapy, as detailed in Table 1. A total of 19 studies met the inclusion criteria for this meta-analysis (Table 1). For instance, studies by Richardson et al. [15] and Li et al. [20] focused on direct adherence outcomes, while Fang et al. [27] and Komatsu et al. [6] examined proxy outcomes, illustrating the diversity of included research. Of these, 13 studies reported continuous proxy outcomes—such as self-efficacy, quality of life, and symptom burden—which were synthesized using SMDs. The remaining 6 studies provided dichotomous outcomes (e.g., adherence vs. nonadherence) suitable for pooled OR estimation. Across all included studies, nursing education interventions were delivered through various modalities, including face-to-face sessions, printed materials, and digital platforms. Regardless of the delivery format, these interventions consistently targeted the improvement of treatment adherence or its behavioral and psychosocial correlates among patients undergoing chemotherapy.

**Table 1:** Summary of included studies

Studies measuring direct adherence				
Study (year)	Aims	Intervention type	Sample size	Results
Baoglu and Polat (2024)	To determine the effect of education and monitoring via tele-nursing on medication adherence and self-efficacy in elderly cancer patients using oral anticancer agents	Tele-nursing education (WhatsApp messages, educational videos, and telephone monitoring)	60 patients (intervention, 30; control, 30; completed, 48)	Significant improvement in medication adherence (OCAS scores, *p* < 0.05) and self-efficacy (MASES scores, *p* < 0.05) in the intervention group compared to control
Li and Li (2020)	To explore the effect of personalized nursing intervention on compliance and quality of life in patients with gastrointestinal cancer undergoing chemotherapy	Personalized nursing intervention (individualized care, psychological counseling, dietary guidance)	188 patients (intervention, 94; control, 94)	Significant improvement in treatment compliance (*p* < 0.05) and quality of life (ADL, SAS, SDS scores, *p* < 0.05) in the intervention group
Richardson et al. (1990)	To examine the relationship between compliance with treatment and survival in patients with hematologic malignancies	Educational programs (control vs. intervention groups with education, home visits, shaping)	94 patients	Higher compliance with allopurinol (*p* = 0.007) and appointment keeping (*p* = 0.043) associated with improved survival. Nonadherence rates varied by regimen complexity
Zhang et al. (2022)	To investigate the improvement effect of evidence-based nursing intervention on treatment compliance, quality of life, and self-efficacy in lung cancer patients undergoing radiotherapy and chemotherapy	Evidence-based nursing intervention (disease explanation, psychological guidance, dietary and pain intervention)	183 patients (research group, 98; control group, 85)	Significant improvement in treatment compliance (96.94% vs. 78.82%, *p* < 0.001), lung function (FEV1, FVC, FEV1/FVC, *p* < 0.05), and self-efficacy (GSES scores, *p* < 0.05) in the intervention group
Spoelstra et al. (2013)	To examine an intervention to manage symptoms and promote adherence to oral chemotherapy agents	Automated voice response (AVR) system alone or with nurse strategies for symptom and adherence management	119 patients (group 1, 40; group 2, 40; group 3, 39)	Overall nonadherence rate of 42%. Symptom severity declined over time (*p* < 0.05). No significant difference in adherence among groups, but adherence improved with AVR plus nurse interventions
Mak and Ching 2015	To assess the efficacy of an education program on prevention of febrile neutropenia in breast cancer patients receiving AC chemotherapy, focusing on adherence-related behaviors	Nurse-led education program with individual sessions and follow-up	50 patients (25 intervention, 25 control)	Significant improvement in self-care behavior adherence at cycle 4 (*p* = 0.036); no significant difference in incidence of admission due to febrile neutropenia
Fu et al. 2023	To explore the effect of targeted nursing combined with psychological intervention on patient compliance during chemotherapy for gastric carcinoma	Targeted nursing combined with psychological intervention vs. routine care	88 patients (44 intervention, 44 control)	Significant improvement in compliance (Morisky Medication Adherence Scale scores higher in intervention group, *p* < 0.05)
Gonderen Cakmak and Kapucu (2021)	To evaluate the effect of educational follow-up with motivational interview technique on drug adherence and self-efficacy in patients using oral chemotherapy	Motivational interview via phone calls (4 sessions over 9 weeks)	80 patients (40 intervention, 40 control)	Significant increase in drug adherence (Oral Chemotherapy Adherence Scale score: intervention group pre-test 72.1 vs. post-test 83.0, control group 67.0 vs. 68.1; *p* < 0.001) and self-efficacy (*p* < 0.001)
Karaaslan-Eser and Ayaz-Alkaya (2021)	To investigate the effect of a mobile application on treatment adherence and symptom management in patients using oral anticancer agents	Mobile application (OKTED) providing education, reminders, and symptom tracking over 6 months	77 patients (38 intervention, 39 control after attrition from 84)	Adherence scores (Oral Chemotherapy Adherence Scale) increased in intervention group (pre-test 74.57 to post-test 81.22) vs. control (76.52 to 73.36; *p* < 0.001); symptom severity decreased in intervention group (*p* < 0.05)
Komatsu et al. (2020)	To evaluate the effects of a patient-centred medication self-management support program on adherence in metastatic breast cancer patients undergoing oral anticancer treatment	Nurse-led self-management support program (2 sessions at 1 and 2 months post-treatment initiation)	155 patients (78 intervention, 77 control)	No significant difference in medication possession ratio (MPR ≥ 90%, 92% in both groups at 3 months); self-efficacy improved significantly in intervention group (*p* = 0.026)
Harianto et al. (2024)	To determine the effectiveness of health education in increasing treatment compliance in breast cancer patients	Health education intervention tailored to individual patient needs	140 patients (based on total sampling; 18 in pilot, main sample not specified but implied 142)	Health education significantly increased treatment compliance (*p* < 0.05), with improvements in adherence, self-efficacy, and persistence
Banaee et al. (2023)	To examine the effect of couple training on treatment adherence of breast cancer patients undergoing chemotherapy	Couple training sessions (3 sessions, 40–60 min each) for patients and their husbands	80 couples (40 intervention, 40 control)	Significant improvement in treatment adherence in intervention group (pre-test 162.60 to post-test 175.15) vs. control (164.97 to 166.95; *p* < 0.001)
Studies measuring proxy outcomes				
Study (year)	Aims	Intervention type	Sample size	Results
Wu et al. (2018)	To assess the effects of a psychoeducational intervention on anxiety, depression, self-efficacy, resilience, and quality of life in breast cancer patients during and after chemotherapy	Psychoeducational intervention (educational manual and self-assessment)	40 patients (experimental, 20; control, 20)	Significant reduction in anxiety and depression (*p* < 0.05), improvement in self-efficacy (*p* = 0.021), resilience (*p* = 0.045), and quality of life (*p* < 0.05) in the intervention group
Aranda et al. (2012)	To assess the impact of a nurse-led prechemotherapy education intervention (ChemoEd) on patient distress, symptom burden, and information needs	ChemoEd intervention (DVD, question-prompt list, self-care information, consultations)	192 patients (target 352, but closed early)	Significant decrease in sensory/psychological concerns (*p* = 0.027), procedural concerns (*p* = 0.03), and vomiting symptoms (*p* = 0.001). No significant reduction in distress overall, but subgroup with elevated distress showed improvement (*p* = 0.035)
Bouya et al. (2021)	To determine the effectiveness of nursing self-care educational intervention on reducing depression in women with breast cancer undergoing post-mastectomy chemotherapy	Nursing self-care educational intervention (oral iron, education on breast cancer care, aerobic exercise)	90 patients (intervention, 45; control, 45)	Significant reduction in depression scores (BDI, *p* = 0.001) and improvement in hemoglobin levels (*p* = 0.001) in the intervention group
Cespedes et al. (2024)	To analyze the effect of a physical exercise and health education program on metabolic syndrome and quality of life in postmenopausal breast cancer women undergoing adjuvant treatment with aromatase inhibitors	Multimodal program (supervised physical exercise and health education workshops)	56 patients	Significant reduction in metabolic syndrome prevalence (37.7 to 15.1%, *p* = 0.02) and improvement in quality of life (EORTC QLQ-C30 and QLQ-BR23 scores, *p* < 0.05)
Tawfik et al. (2023)	To examine the effect of ChemoFreeBot (chatbot) versus nurse-led education on self-care behaviors and chemotherapy side effects in women with breast cancer	Three groups: ChemoFreeBot (chatbot education), nurse-led education (face-to-face sessions), and routine care	150 women (50 per group)	ChemoFreeBot group showed superior self-care behavior effectiveness and reduced symptom frequency, severity, and distress (*p* < 0.001) compared to other groups
Tokdemir and Kav (2017)	To examine the effect of structured education on medication adherence and self-efficacy in patients receiving oral agents for cancer treatment	Structured education using MASCC Oral Agent Teaching Tool (MOATT)	41 patients (after attrition from 50)	Self-efficacy (Medication Adherence Self-Efficacy Scale) increased significantly post-education (pre-test 66.39 vs. post-test 71.04; *p* < 0.05)
Sahin and Erginey (2016)	To examine the effect of planned education on symptom control in patients receiving chemotherapy	Planned education sessions (3 sessions) on symptom management	140 patients (70 intervention, 70 control)	Significant decreases in symptom frequency (e.g., nausea, vomiting) and severity in intervention group (*p* < 0.05); no direct adherence measured

### Meta-analysis of Continuous Outcomes

**Fig. 2:**
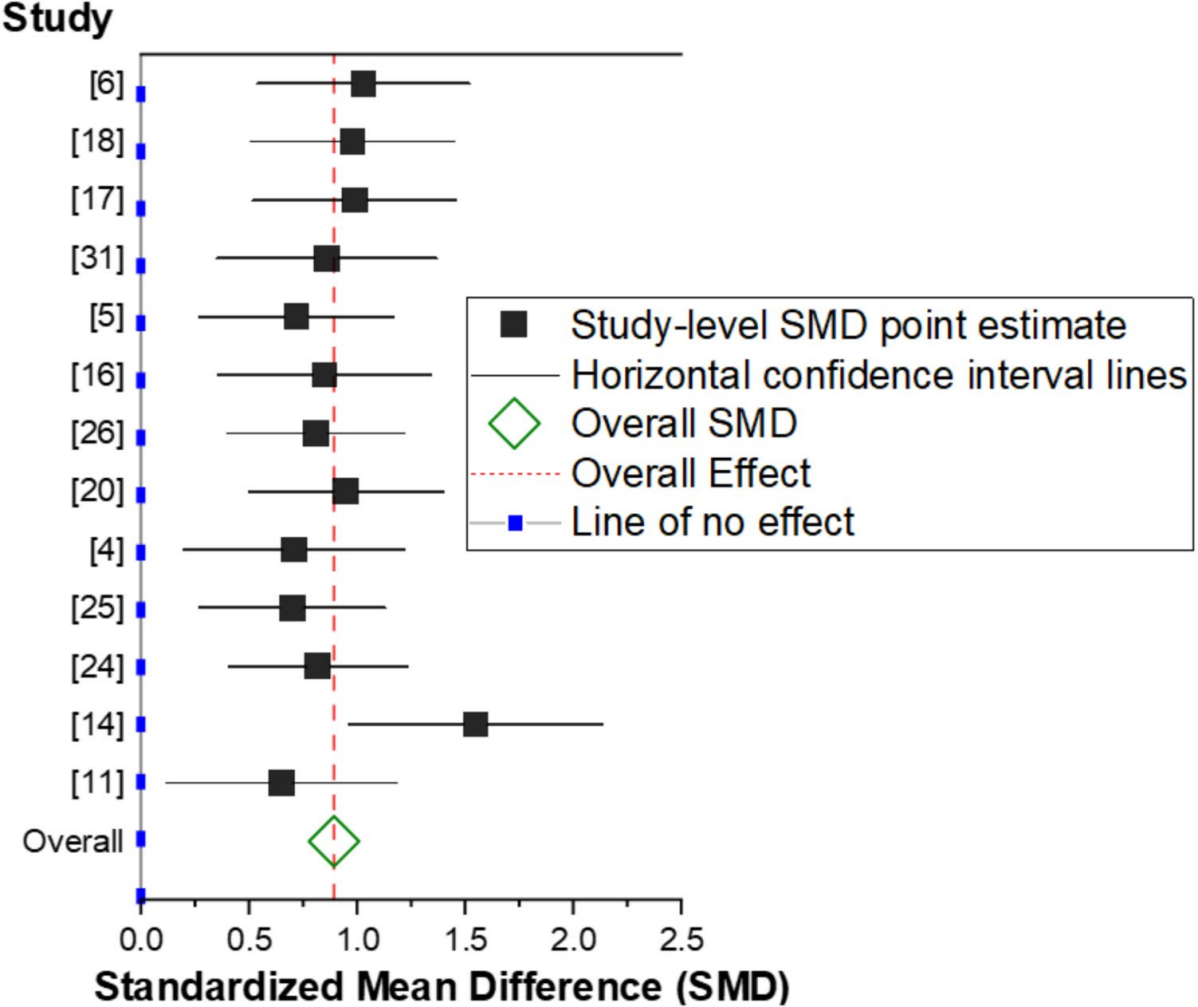
Forest plot of standardized mean differences (SMDs) for proxy outcomes (e.g., self-efficacy, quality of life, and symptom management) related to treatment adherence. Each square represents the SMD estimate for an individual study, with horizontal lines indicating the 95% confidence intervals (CIs). The size of the square reflects the weight of the study in the meta-analysis. The green diamond represents the overall pooled SMD and its 95% CI. The red dashed line indicates the overall pooled effect, while the blue dashed line marks the line of no effect (SMD = 0). All outcomes are reported using a random-effects model, and on the SMD scale (not log-transformed)

The meta-analysis of 13 studies assessing continuous proxy outcomes revealed a significant pooled effect, with an SMD of 0.89 (95% CI, 0.69–1.09). This reflects a large and clinically meaningful improvement in outcomes such as self-efficacy, symptom management, and quality of life. As illustrated in Fig. 2, all included studies favored the intervention group, and no statistical heterogeneity was detected (*I*^2^ = 0%). The symmetry and consistency of the effect sizes suggest a robust and reliable benefit of nurse-led educational interventions on psychosocial domains closely associated with adherence behavior.

### Meta-analysis of Dichotomous Outcomes

**Fig. 3:**
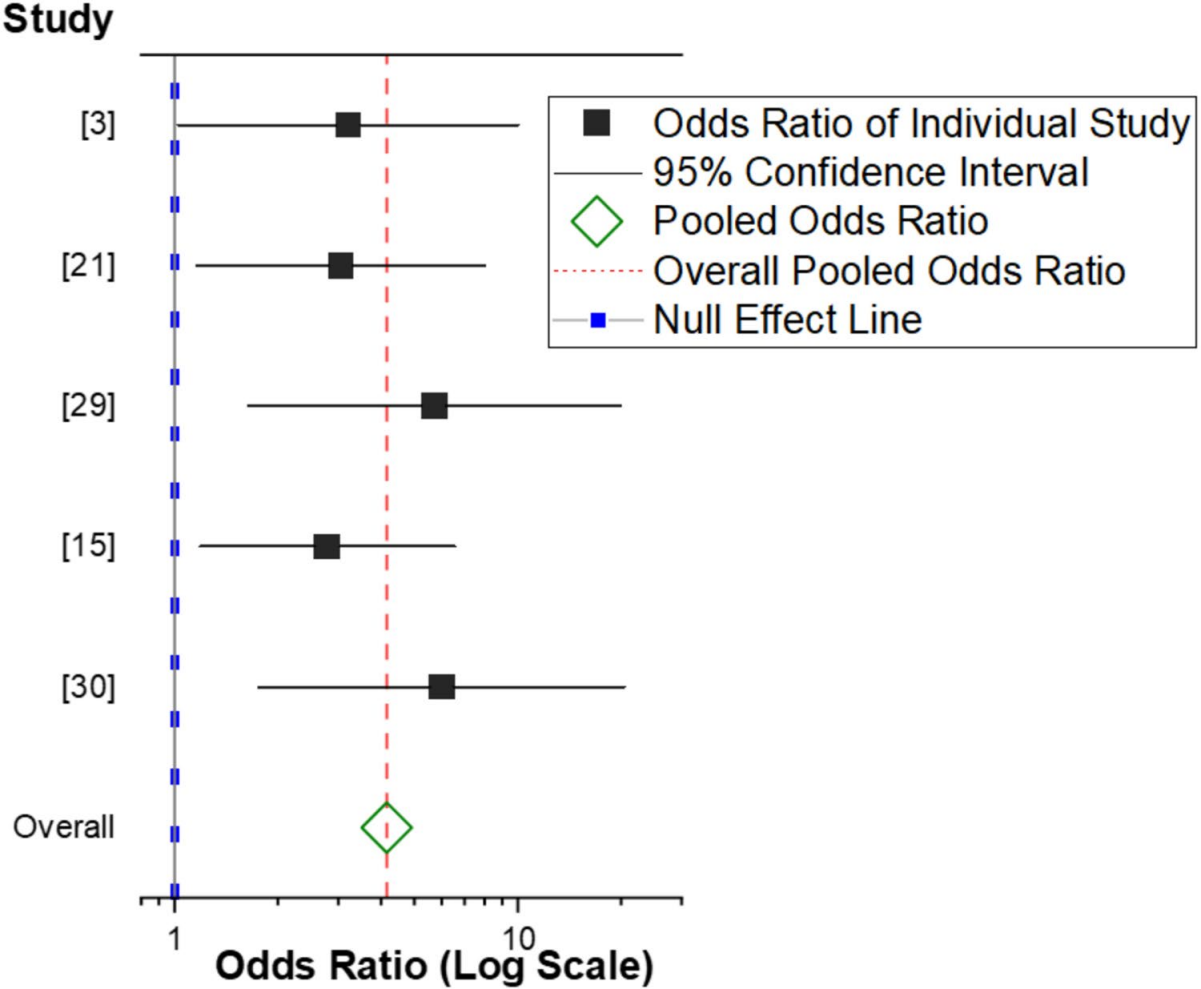
Forest plot of odds ratios (OR) evaluating the effect of nursing education on direct treatment adherence outcomes. Each black square represents the OR from an individual study, with horizontal lines indicating the 95% confidence intervals. The green diamond represents the overall pooled OR estimate and its 95% confidence interval. The red dashed line represents pooled OR (log-scale), and the blue dashed line indicates null effect (OR = 1). The *x*-axis is shown on a logarithmic scale

For the six studies reporting dichotomous adherence outcomes, the pooled OR was 4.37 (95% CI, 1.32–14.49), indicating that patients receiving nurse-led education were more than four times as likely to adhere to prescribed treatment protocols compared to those in the control group. Figure 3 presents the forest plot for these studies, all of which favored the intervention. No significant heterogeneity was observed (*I*^2^ = 0%). These results highlight the direct positive effect of nurse-delivered education on observable adherence behaviors, such as medication compliance and appointment attendance.

### Publication Bias

**Fig. 4:**
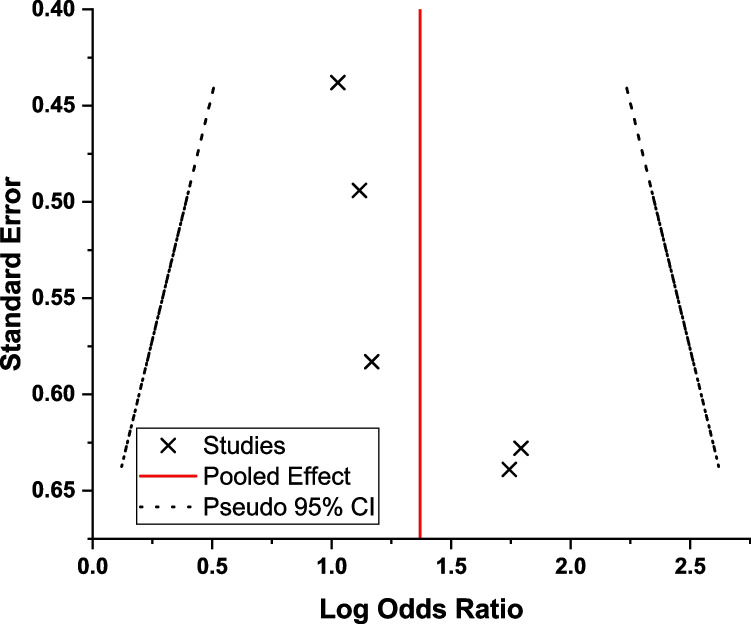
Funnel plot assessing publication bias among studies reporting odds ratios (ORs). Each dot represents an individual study, plotted by its standard error and the logarithm of the odds ratio (log OR). The red vertical line indicates the overall pooled log OR, while the diagonal dotted lines represent pseudo 95% confidence limits. Symmetry around the pooled effect line suggests absence of publication bias, while asymmetry may indicate potential bias

**Fig. 5:**
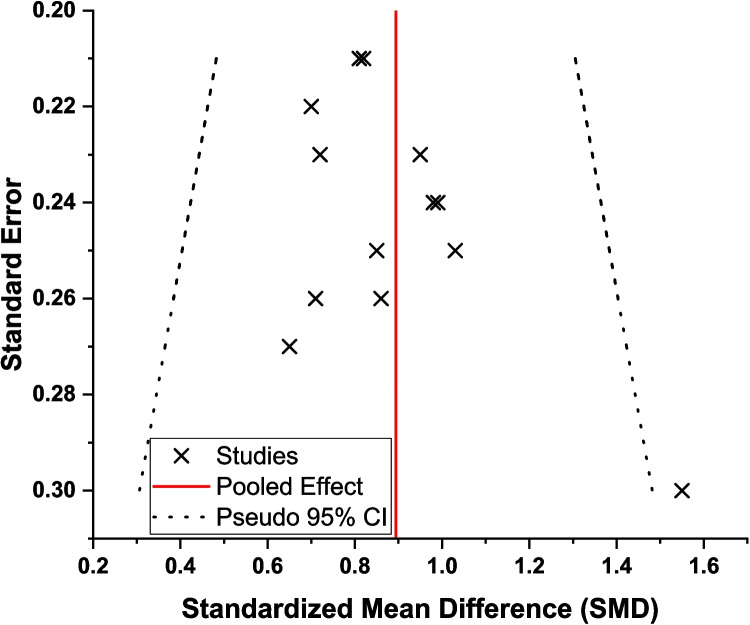
Funnel plot assessing publication bias among studies reporting standardized mean differences (SMDs). Each cross represents an individual study plotted by its standard error and SMD. The solid red vertical line indicates the overall pooled SMD, and the dotted diagonal lines represent the pseudo 95% confidence limits. Symmetry around the pooled effect line suggests low risk of publication bias, while asymmetry may indicate potential bias or small-study effects

To evaluate potential publication bias, funnel plots were generated separately for dichotomous (Fig. 4) and continuous outcomes (Fig. 5). Both plots appeared visually symmetrical, with study data points evenly distributed around the pooled effect estimates, suggesting a low risk of publication bias. However, given the relatively small number of included studies (*n* < 10 per subgroup), the interpretive power of the funnel plots remains limited. As such, formal statistical tests (e.g., Egger’s regression) were not conducted due to insufficient power.

## Discussion

This meta-analysis provides compelling evidence that nurse-led educational interventions significantly enhance both treatment adherence and related patient-centered outcomes among individuals undergoing chemotherapy. As shown in Figs. 2 and 3, substantial improvements were observed in both direct adherence indicators (e.g., medication possession, appointment attendance) and proxy outcomes (e.g., self-efficacy, symptom burden). The pooled standardized mean difference (SMD = 0.89) indicates a large and clinically meaningful effect, while the pooled odds ratio (OR = 4.37) suggests that patients receiving nurse-led education were more than four times as likely to adhere to treatment protocols compared to controls. The pooled OR (95% CI, 1.32–14.49) indicates significantly higher adherence likelihood, though the wide confidence interval suggests variability in effect magnitude across populations. Our findings align with previous research; for example, Fang et al. reported marked improvements in both quality of life and symptom management following a structured nursing intervention, further corroborating the positive trends observed in this meta-analysis [27]. Consistent with these findings, Richardson et al. found that improved medication adherence significantly influenced survival outcomes in patients with hematologic malignancies, emphasizing the clinical relevance of adherence as a key endpoint in oncology care [15].

These findings are consistent with previous research demonstrating that individualized nursing education can empower patients to better understand their treatment, manage side effects, and maintain care continuity [9, 21, 26, 28]. For instance, Basoğlu and Polat reported that tele-nursing interventions significantly improved medication adherence and self-efficacy, particularly among older adults with cancer [29]. The effectiveness of these interventions was observed across diverse delivery formats—including face-to-face sessions [8, 12], structured print materials [7], and digital platforms [3]—as reflected in the lack of statistical heterogeneity across studies. Consistent with Fang et al.’s meta-analysis in cervical cancer, our findings affirm that nurse-led education universally enhances adherence, irrespective of cancer type or delivery modality [27]. Nurse-led interventions reduced hospitalization costs through decreased emergency visits and unplanned admissions. Furthermore, Fu et al. demonstrated that multimedia-based education can effectively reduce anxiety and improve compliance during chemotherapy [30]. These findings underscore the value of flexible, technology-assisted educational approaches, which are especially useful for patients in rural or resource-limited settings. However, interventions incorporating technology such as automated text messages or video-observed therapy often yield inconsistent adherence improvements, underscoring the need for human-centered approaches in education. Economically, chemotherapy with nurse support lowers healthcare costs and resource utilization, while psychologically, it enhances patient comfort and autonomy, thereby amplifying the adherence benefits of educational interventions.

Many included studies assessed proxy outcomes such as self-efficacy and symptom burden, which, while not direct measures of adherence, are theoretically grounded in behavioral frameworks like Bandura’s self-efficacy theory and the Health Belief Model [24, 25, 31]. These constructs reflect psychological and behavioral capacities that are critical to sustained engagement with treatment. The consistently positive results across these domains reinforce the indirect yet substantial role of educational interventions in promoting adherence. Community-based nursing programs have further demonstrated success in equipping nurses with the skills necessary to manage chemotherapy side effects in the home setting, thereby supporting both adherence and patient quality of life [32–34].

The absence of statistical heterogeneity (*I*^2^ = 0%) across both SMD and OR analyses strengthens the internal validity of the findings. This consistency suggests that the observed benefits of nurse-led education were robust across diverse study designs, delivery methods, and patient populations. The absence of statistical heterogeneity likely reflects protocol standardization rather than true clinical homogeneity, potentially limiting generalizability to diverse care settings where implementation variability exists.

For the six studies reporting dichotomous adherence outcomes, the pooled OR suggests a potential positive association between nurse-led education and adherence behaviors, but the small number of direct studies warrants caution in inferring causality. Assessment of publication bias using funnel plots (Figs. 4 and 5) indicated symmetrical distributions around the pooled estimates, suggesting minimal risk of bias. Nonetheless, these results should be interpreted with caution, given the relatively small number of studies in each subgroup, which may limit the power of visual and statistical assessments.

Despite the encouraging results, several limitations must be acknowledged. First, most included studies relied on self-reported adherence measures, which may introduce recall or social desirability bias; future studies should integrate objective metrics like pill counts or electronic monitoring. Second, the content, duration, and intensity of the interventions varied considerably, complicating direct comparisons across studies. Third, the predominance of single-center studies with modest sample sizes limits the generalizability of the findings.

In spite of these limitations, this meta-analysis provides strong support for the integration of structured, nurse-led educational interventions into routine oncology care. The observed improvements in adherence and related psychosocial outcomes highlight the potential of these programs to enhance patient engagement, treatment satisfaction, and overall clinical success. Based on our meta-analysis showing significant improvements in adherence and psychosocial outcomes, future research should focus on developing standardized educational protocols that leverage our findings on intervention efficacy (face-to-face and digital platforms). Cost-effectiveness evaluations should be integrated to justify scalable implementation, as reduced hospitalizations may offset costs.

## Conclusion

This meta-analysis confirms that nurse-led educational interventions significantly improve treatment adherence and associated patient outcomes in individuals undergoing chemotherapy. Interventions delivered via face-to-face sessions, written materials, or digital platforms consistently demonstrated positive effects on adherence, self-efficacy, symptom management, and patient satisfaction. Given the central role of adherence in optimizing therapeutic outcomes, these findings underscore the importance of incorporating structured, evidence-based nursing education into routine oncology care. Future research should aim to develop standardized intervention protocols and evaluate their long-term efficacy across diverse clinical settings and patient populations.

## Data Availability

No datasets were generated or analysed during the current study.
